# Socio-Demographic, Nutritional, and Lifestyle Factors Influencing Perceived Sleep Quality in Spain, with a Particular Focus on Women and Young People

**DOI:** 10.3390/nu17061065

**Published:** 2025-03-18

**Authors:** Elena Sandri, Agnese Broccolo, Michela Piredda

**Affiliations:** 1 Faculty of Medicine and Health Sciences, Catholic University of Valencia San Vicente Mártir, c/Quevedo, 2, 46001 Valencia, Spain; 2 Doctoral School, Catholic University of Valencia San Vicente Mártir, c/Quevedo, 2, 46001 Valencia, Spain; 3 Department of Biomedicine and Prevention, Tor Vergata University, Via Montpellier, 00133 Rome, Italy; agnese.broccolo@alumni.uniroma2.eu; 4 Research Unit Nursing Science, Department of Medicine and Surgery, Campus Bio-Medico di Roma University, Via Alvaro del Portillo, 21, 00128 Rome, Italy

**Keywords:** sleep duration, sleep quality, sleep hygiene, diet, food and nutrition, physical activity, social habits, socio-demographic factors

## Abstract

**Objectives:** This study examines the relationship between nutrition, lifestyle habits, and perceived sleep quality in a cross-sectional analysis of 22,181 Spanish adults. **Methods:** Data were collected between August 2020 and November 2021 using the Nutritional and Social Healthy Habits (NutSo-HH) questionnaire, which assessed variables such as sleep duration, self-perceived restfulness, dietary patterns, and physical activity. **Results:** Findings indicate that 48.9% of participants sleep 7–8 h per night, while 8.6% sleep less than 6 h. Approximately 50% report frequently feeling rested, whereas 45.4% seldom or sometimes feel rested. Non-parametric Mann–Whitney and Kruskal–Wallis tests with Dwass–Steel–Critchlow–Fligner (DSCF) correction revealed that perceived sleep quality had an average score of 3.39 on a 0–5 scale, with significant differences based on socio-demographic and lifestyle factors (*p* < 0.001 for sex, age, education, income, and living in a family). Participants with sufficient sleep reported a lower BMI, a higher nutritional index, and more weekly physical activity. A network analysis demonstrated strong clustering between sleep variables and eating behaviors. Although causality cannot be established in this observational study, the results suggest that better sleep is associated with the lower consumption of sugary drinks and ultra-processed foods, as well as improved body image and mental health. **Conclusions:** These findings highlight the interconnectedness of sleep, nutrition, and lifestyle habits, suggesting that targeted interventions in any of these areas could positively influence the others, ultimately improving overall health outcomes.

## 1. Introduction

Sleep is not a passive, uniform, or immutable phenomenon but rather a dynamic, structured, and active process. From a behavioral perspective, it manifests as a state characterized by reduced mobility, closed eyes, and temporary loss of consciousness, from which an individual can be awakened by appropriate sensory stimuli [1].

The amount of sleep required varies among individuals and changes throughout the life cycle [2]. The American Academy of Sleep Medicine provides recommendations on the optimal duration of sleep at different life stages. Newborns require 16–18 h of sleep per day, preschool children need 11–12 h, school-age children and adolescents require approximately 10 h, and most adults need 7–8 h [3].

During sleep, significant physiological changes occur, including neurotransmitter release, immune system modulation, and metabolic adjustments [4]. Sleep follows a cyclical pattern characterized by rapid eye movement (REM) and non-rapid eye movement (NREM) sleep, both of which are essential for cognitive function, physical recovery, and immune regulation [5,6,7]. REM sleep supports memory consolidation and information processing, while NREM sleep plays a key role in tissue repair and immune-system maintenance [8].

Research indicates that sleep disturbances are linked to various health conditions, including neurodegenerative diseases such as Alzheimer’s, as well as metabolic and cardiovascular disorders [9,10,11,12,13]. Chronic sleep deprivation adversely affects stress management, metabolic balance, and reproductive health [14]. Additionally, multiple factors influence sleep quality, including physiological variations, environmental conditions, emotional states, and cognitive functions, making it a key determinant of overall well-being [10].

Subjective sleep perception plays a crucial role in assessing sleep quality and is closely related to individual well-being, regardless of clinical evaluations [15,16]. While polysomnography and actigraphy provide objective measures of sleep structure and duration, individuals with insomnia often misperceive their sleep, underestimating their total sleep time and overestimating wakefulness [17]. Given the strong connection between well-being and sleep perception, evaluating subjective sleep quality is essential for a comprehensive understanding of sleep health [18,19].

Although the significance of sleep for overall well-being is well recognized, a substantial gap remains in the literature regarding the relationship between subjective sleep perception and socio-demographic, dietary, and lifestyle factors. While many studies focus on the clinical aspects of sleep, research exploring the influence of daily habits and personal perceptions of rest is comparatively limited [20,21].

This study aims to address gaps in the literature by examining the relationship between subjective sleep quality and socio-demographic, dietary, and lifestyle factors. By emphasizing subjective sleep perception, it acknowledges its critical role in shaping overall health experiences. Integrating data on sleep quality, dietary habits, and lifestyle factors, this study provides a broader perspective on its potential determinants. This approach not only bridges existing research gaps but also lays the foundation for targeted interventions to enhance sleep quality and overall well-being.

### 1.1. Study Hypotheses

Sleep quality is a fundamental component of psychophysical well-being, including socio-demographic characteristics, dietary habits, lifestyle choices, and physical activity. Research indicates that variables such as age, gender, education level, daily behaviors, and physical activity impact sleep perception [22,23,24,25,26].

Based on existing evidence, we hypothesize that subjective sleep perception is shaped by an interplay of socio-demographic factors, lifestyle choices, and physical activity levels, collectively influencing perceived sleep quality and restfulness.

### 1.2. Study Objective

This study aims to examine how socio-demographic characteristics, dietary habits, and lifestyle factors affect subjective sleep perception. By analyzing these variables, we seek to determine their relative contributions to perceived sleep quality and restfulness. The findings will inform targeted strategies to improve sleep health and overall well-being by addressing modifiable lifestyle factors.

## 2. Materials and Methods

### 2.1. Study Design and Participant Selection

This study was conducted as a cross-sectional analysis involving adult residents of Spain. To ensure result reliability, individuals with chronic illnesses affecting dietary patterns, as well as those experiencing temporary dietary disruptions (e.g., hospitalization or incarceration), were excluded. Additionally, individuals with conditions influenced by nutrition that could affect sleep, such as type 2 diabetes, pregnancy, or digestive diseases, were also excluded.

Moreover, individuals with conditions that could independently alter sleep—such as diagnosed sleep disorders, neurological diseases, or chronic illnesses with nighttime symptoms (e.g., fibromyalgia, chronic pain, or arrhythmias)—were excluded. The final sample included 22,181 participants.

### 2.2. Sample Size Calculation

The required sample size was determined using data from the Continuous Population Statistics (ECP) of the Spanish National Institute of Statistics (INE) [27]. As of 1 January 2020, the population of Spain was 42,105,707. Applying the standard sample size formula for known populations, with a 95% confidence level, a 5% margin of error, and assuming maximum variability (*p* = q = 50%), the minimum required sample was 385 participants.

### 2.3. Ethical Considerations

The study adhered to the ethical principles outlined in the Declaration of Helsinki [28] and received approval from the Research Ethics Committee of the Catholic University of Valencia (approval code UCV/2019-2020/152, 18 June 2020).

Before completing the questionnaire, all participants received detailed information about the study’s objectives. They were assured of the anonymity of their responses and the aggregated nature of data analysis. Proceeding with the questionnaire implied informed consent for data use in the study and publication of findings.

### 2.4. Measurement Tool

Data were collected using the Nutritional and Social Healthy Habits Scale (NutSo-HH) [29], supplemented by socio-demographic questions and qualitative insights.

The NutSo-HH scale was developed and validated in two phases. In the first phase, the conceptual framework was established, followed by the design, cognitive review, expert evaluation, and pilot testing. A panel of seven experts (a nutritionist, two general practitioners, two psychologists, a social educator, and a communication specialist) assessed content validity. Face validity was tested through a pilot study with 53 individuals resembling the target population. In the second phase, the finalized scale was administered to a sample of 571 young adults in Spain, recruited via non-probabilistic sampling through social media. Reliability analysis yielded omega coefficients ranging from 0.521 to 0.815, indicating acceptable internal consistency.

NutSo-HH is a multidimensional instrument comprising 23 items designed to assess various lifestyle factors, including dietary habits, physical activity, sleep patterns, and alcohol consumption. These items are categorized into six primary factors:

F1: Mediterranean Diet
F2: Healthy and Unhealthy Foods
F3: Meat and Dairy Products
F4: Eating Disorders
F5: Sleep Habits
F6: Alcohol Consumption

Additionally, the scale includes two second-order factors:

NUTRI (encompassing F2 and F3)
HH (Healthy Habits, encompassing F4 and F5)

### 2.5. Data Collection

Participants were recruited through both digital and in-person outreach strategies. Initial contact was made via email and social media, with a particular focus on Instagram, where a dedicated account (@elretonutricional) was created for this study. This platform played a key role in engaging health professionals and influencers who helped disseminate the survey within their networks. To further expand participation, researchers leveraged their professional and personal networks on LinkedIn, Twitter, WhatsApp, and Facebook. The survey was also distributed at physical locations with diverse clientele, such as pharmacies and tobacconists, where business owners facilitated on-site sharing. A snowball sampling approach [30] was employed, encouraging initial participants to refer eligible individuals until the target sample size was reached. The study adhered to STROBE guidelines [31], and data collection spanned 15 months, from August 2020 to November 2021.

### 2.6. Variables

The study assessed dietary habits, eating disorders symptoms, and social and lifestyle habits using the 23-item Nutritional and Social Healthy Habits (NutSo-HH) scale. Habit-related variables were categorized following previous research [32,33,34], converting qualitative data into nominal quantitative variables.

A wide range of socio-demographic, anthropometric, and health-related data, including weight, height, and self-perceived health status, were recorded. For analysis, socio-demographic variables were grouped as follows:

Sex: Classified as men or women.

Age: Divided into youth (≤30 years old) and adults (>30 years old).

Education: Categorized into basic education (including individuals with no formal education, primary or compulsory secondary education, and those with baccalaureate or vocational education) and higher education (including those with a university degree, master’s degree, or PhD).

Income level (According to Royal Decree 231/2020 of 4 February, the Minimum Individual Wage (Salario Mínimo Interprofessional: SMI) for 2020 was set at 950 EUR per month, distributed in 14 payments, which is equivalent to 1108 EUR if divided into 12 payments. Considering the economic and social structure of Spain and following the logic that a family needs an income higher than the individual SMI, it can be estimated that a family of four should have an income of at least 2 times the SMI to maintain an adequate standard of living. This is why, for our study, we set the monthly income level per household at 2200 EUR/month [35]): Grouped into low income (household earning less than EUR 2200 gross per month) and medium–high income (households earning more than EUR 2200 gross per month).

Municipality size: Categorized into small municipalities (<2000 inhabitants), medium-sized towns (2000 to 10,000 inhabitants), and cities (>10,000 inhabitants).

Living arrangements: Classified as living alone or not living alone.

Family life: Defined as living with family or without family.

Spanish regions: Participants were grouped based on Spain’s different autonomous communities.

#### 2.6.1. IASE—Spanish Healthy Eating Index

Dietary habits were evaluated using the Spanish Healthy Eating Index (IASE), an adapted version of the validated scale developed by Norte and Navarro [36]. This version includes key dietary categories such as fruits, vegetables, meat, dairy, cereals, legumes, and soft drinks. The index assesses the frequency of the consumption of foods recommended for daily and weekly intake, as well as those meant for occasional consumption. It also incorporates dietary variety, a crucial element of a balanced diet. A scoring system assigns 10 points to behaviors that align with the Spanish Society of Community Nutrition (SENC) guidelines [37], with a maximum possible score of 73. Participants’ eating habits were classified into three categories based on their IASE score:

Healthy: 58.4–73
Needs Changes: 36.5–58.4
Unhealthy: <36.5

#### 2.6.2. Sleep Variables

A categorization approach, consistent with previous research [32,33,34], was applied to convert qualitative sleep-related data into nominal quantitative variables. Sleep variables were classified as follows:

- Sleeping hours: <6 h = 1; 6–7 h = 2; 7–8 h = 3; >8 h = 4
- Getting up rested: Never = 1; Very seldom/sometimes = 2; Frequently/almost always = 3; Always = 4
- Sleep quality: Rated on a Likert scale from 0 (worst) to 5 (best).

To further analyze the relationship between nutrition, lifestyle variables, and perceived sleep quality, sleep variables were grouped for clarity:

- Sleeping hours:
  ∘ Insufficient Sleep Hours (ISH): <6 h or 6–7 h
  ∘ Sufficient Sleep Hours (SSH): 7–8 h or >8 h
- Getting up rested:
  ∘ NO Get Up Rested (NO_GUR): Never, very seldom, or sometimes
  ∘ YES Get Up Rested (YES_GUR): Frequently, almost always, or always
- Sleep quality:
  ∘ Low Sleep Quality (LSQ): Scores of 0 or 1
  ∘ Medium Sleep Quality (MSQ): Scores of 2 or 3
  ∘ High Sleep Quality (HSQ): Scores of 4 or 5

### 2.7. Data Analysis

The dataset was initially processed to remove erroneous or atypical entries, including inaccurate responses and extreme outliers in height and weight. Participants with a BMI below 14 or above 40 were excluded. After this data-cleaning step, the Shapiro-Wilk test was conducted to assess normality, and the results showed that none of the variables followed a normal distribution. This finding was further confirmed by the Q-Q plots. [38].

Given the non-normal distribution, different statistical tests were applied based on the variable type. Chi-square tests were used for categorical variables, while the non-parametric Mann–Whitney U test was employed for comparisons between two independent groups. The Kruskal–Wallis test was used to compare three or more independent groups of ordinal and continuous variables. To enhance the robustness of the findings, the Dwass–Steel–Critchlow–Fligner (DSCF) method was applied for post hoc pairwise comparisons following the Kruskal–Wallis test. This method, unlike the Bonferroni correction, balances Type I error control with sufficient sensitivity, making it more effective for detecting meaningful differences between groups.

Categorical variables are presented as counts and percentages, while continuous variables are reported as means and standard deviations. A significance level of 0.05 was applied to all statistical analyses.

To explore the relationships between health, lifestyle, and sleep variables in greater depth, a network analysis was conducted. In this representation, nodes (circles) denote individual variables, while edges (connecting lines) illustrate relationships or correlations. Edge thickness and color visually depict the strength and direction of these associations.

Spearman’s correlation coefficients determined edge weights. To isolate direct associations and mitigate spurious correlations, we applied the Gaussian Graphical Model (GGM) with the Extended Bayesian Information Criterion (EBIC) model selection [39]. This method estimates partial correlations while controlling for other variables, ensuring a more precise depiction of the relationships.

The EBIC model introduces regularization, improving network sparsity and stability while minimizing overfitting. This approach highlights meaningful associations, making complex relationships easier for both experts and non-experts to interpret.

All statistical analyses were conducted using Jamovi (Version 2.3.28.0). Gaussian networks were generated in Jamovi, while additional graphical visualizations were created using Microsoft Excel.

## 3. Results

### 3.1. Sample Description

**Figure 1:**
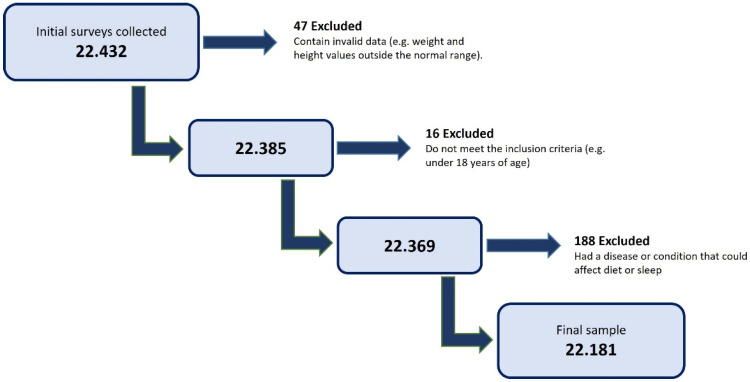
Flow chart for obtaining the final validated sample.

Figure 1 illustrates the inclusion and exclusion process, detailing the steps from the initial sample collection to the final validated dataset.

Table 1 provides an overview of the sample’s socio-demographic profile. The majority of participants were women (80.8%), with a mean age of 34.9 years. Regarding education, 68.3% held a bachelor’s degree or higher. Income distribution was relatively balanced, with 43.9% classified as low-income and 47.9% as medium–high income.

**Figure 2:**
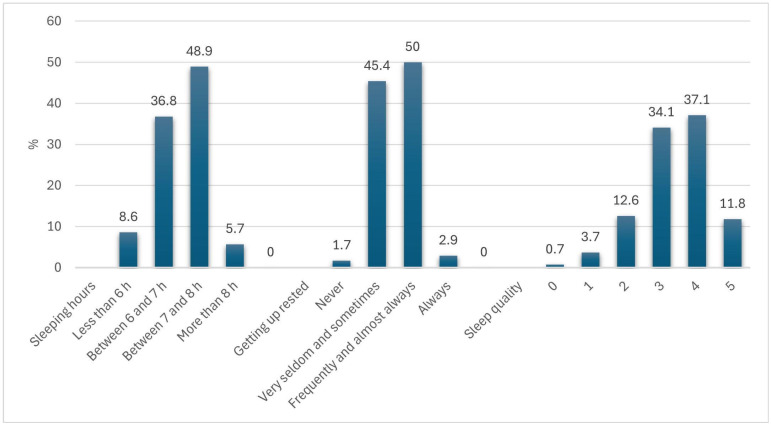
Sleep duration, sleep quality and feeling of getting up rested.

Sleep duration varied among participants: 48.9% reported sleeping 7–8 h, 36.8% slept 6–7 h, while fewer slept less than 6 h (8.6%) or more than 8 h (5.7%). Regarding restfulness, 50% frequently or almost always woke up feeling rested, while 45.4% reported feeling rested seldom or sometimes. Sleep quality was predominantly rated as 3 (34.1%) or 4 (37.1%) on a 0–5 scale, with a mean score of 3.39 (SD = 1.02). Figure 2 presents a detailed breakdown of these results.

### 3.2. Socio-Demographic Differences in Sleep Variables

Table 2 presents variations in sleep patterns across socio-demographic groups. Women generally sleep slightly longer than men but report feeling less rested and experiencing lower sleep quality, with statistically significant differences. Younger adults (18–30 years) tend to sleep longer and rate their sleep quality better. Education level shows a minor positive correlation with sleep quality, while income primarily influences feelings of restfulness and overall sleep quality, with higher-income individuals reporting better outcomes. Municipality size and living arrangements exhibit weak associations with sleep variables, whereas family life has a slight positive effect on sleep quality. Although some of these differences are statistically significant, their practical impact remains moderate due to small absolute values.

### 3.3. Relationship Between Nutrition, Lifestyle, and Sleep Variables

Table 3a,b highlight statistically significant differences across several variables based on sleep duration (Insufficient Sleep Hours [ISH] vs. Sufficient Sleep Hours [SSH]) and the perception of waking up rested (NO Get Up Rested [NO_GUR] vs. YES Get Up Rested [YES_GUR]). While these differences are statistically significant, their absolute values remain relatively small.

Participants with SSH had higher IASE scores (*p* < 0.001) and self-perceived health ratings (*p* < 0.001) than those with ISH. They also had a lower Body Mass Index (BMI) (*p* < 0.001), consumed fewer sugary soft drinks (*p* < 0.001), and smoked less (*p* < 0.001). Additionally, they engaged in more weekly sports activities (*p* < 0.001) and exhibited slightly lower levels of obesophobia and lack of self-control (*p* < 0.001).

Similarly, participants in the YES_GUR group showed better adherence to the IASE (*p* < 0.001), lower BMI (*p* < 0.001), and higher fish consumption (*p* < 0.001). They also rated their health more positively (*p* < 0.001), engaged in more sports (*p* < 0.001), and had lower levels of obesophobia and lack of self-control (*p* < 0.001).

Notably, differences in fast food, ultra-processed food, juice, and alcohol consumption were minimal or non-significant. Similarly, habits like excessive alcohol intake and night outings were comparable, with only marginal variations.

### 3.4. Analysis of the Relationship Between Nutrition, Lifestyle Variables, and Sleep Quality

Table 4 compares nutrition and lifestyle variables across three sleep quality groups: low (LSQ), medium (MSQ), and high (HSQ). Overall, better sleep quality is associated with healthier habits and improved physical and psychological well-being.

IASE scores increase consistently with better sleep (LSQ: 52.0, MSQ: 55.0, HSQ: 56.0), while Body Mass Index (BMI) decreases (LSQ: 24.0, MSQ: 23.2, HSQ: 22.8), highlighting a clear link between sleep and healthier body weight. Dietary patterns also show significant trends—fried food consumption decreases slightly, with significant differences between LSQ and HSQ, while fast food and ultra-processed food intake decline significantly as sleep quality improves. Fish and water consumption increase modestly, with statistically significant differences in most comparisons. Sugary soft drink consumption drops significantly, while juice consumption shows minimal changes. Coffee and energy drink consumption remains stable across groups.

Lifestyle patterns further support these findings. Although a sedentary lifestyle becomes less common with better sleep, the change is not statistically significant. However, self-perceived health improves significantly across all groups, and physical activity levels, measured by minutes of sport per week, increase significantly. Smoking rates decrease significantly with better sleep, while alcohol consumption rises slightly but significantly. Interestingly, behaviors like excessive alcohol consumption remain unchanged across sleep groups. Night outings increase slightly with better sleep, showing significant differences between groups. Psychological factors also play a role—obesophobia, lack of self-control, and body image dissatisfaction decrease significantly as sleep quality improves, reinforcing the connection between better sleep and enhanced mental health and self-perception.

### 3.5. Network Analysis of Health, Lifestyle, and Sleep Variables

**Figure 3:**
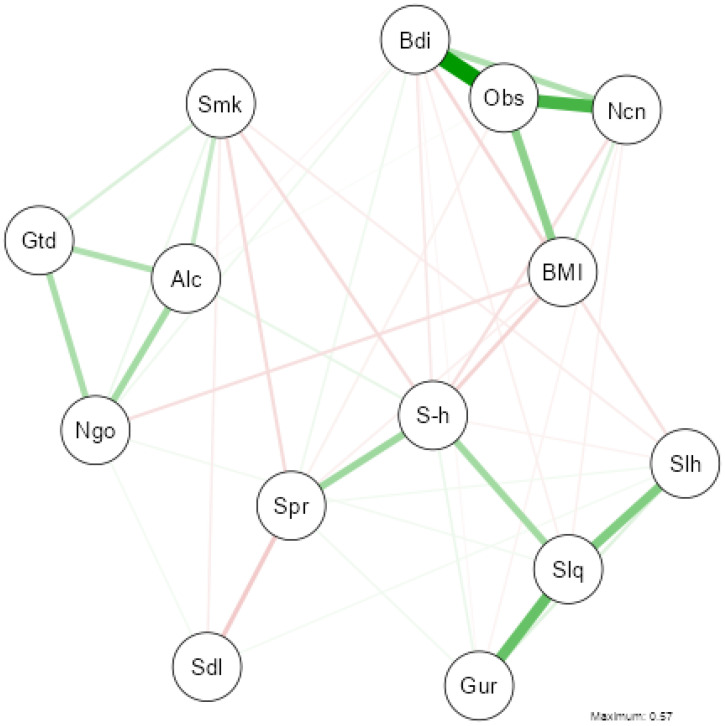
Gaussian Graphical Model of the network analysis between health, lifestyle and sleep variables. (NOTE: The thickness of the lines reflects the magnitude of the relationship (partial correlation), while the colour indicates the direction: green for positive relationships and red for negative relationships).

The network analysis (Figure 3) reveals significant relationships among lifestyle and sleep variables, identifying key clusters and associations. A strong cluster, indicated by thick green edges, includes “Bdi” (Body image), “Obs” (Obesophobia), “BMI”, and “Ncn” (No Control), suggesting a strong positive association among these variables. This highlights their central role in body image and weight-related concerns.

Similarly, “Slh” (Sleeping hours), “Slq” (Sleep quality), and “Gur” (Getting up rested) are closely connected, reflecting their strong interdependence. Another cluster comprises “Alc” (Alcohol), “Smk” (Smoking), and “Ngo” (Night Outings), indicating a pattern of social behaviors associated with nightlife and substance use. “Spr” (Sport) exhibits weaker associations with other variables, suggesting its relative independence from body image and sleep-related clusters while still being linked to general lifestyle habits.

**Figure A1:**
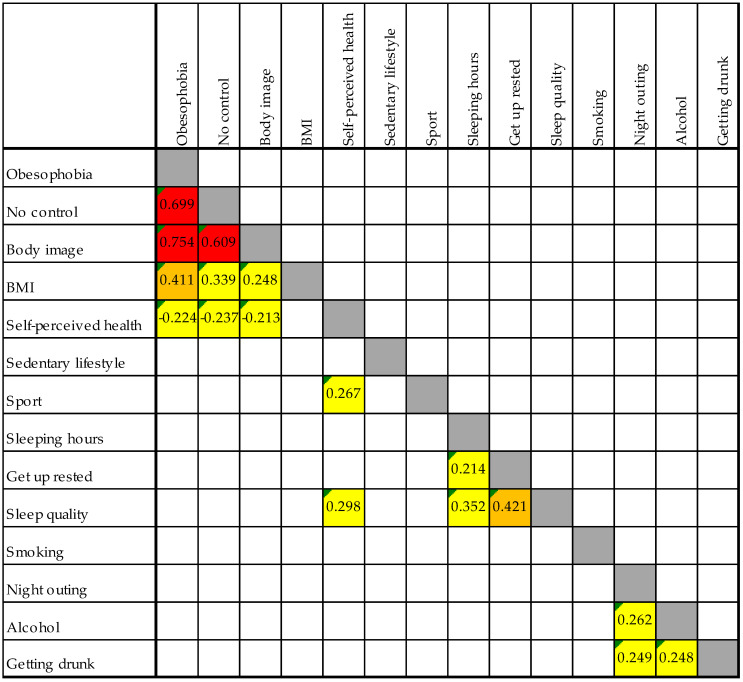
Spearman’s correlation for each pair of lifestyle and sleep variables (White cells have no correlation (0.00–0.19); Yellow = Low correlation (0.20–0.39); Orange = Moderate correlation (0.40–0.59); Red = Strong correlation (≥0.60); Grey cells corresponding to the diagonal of the matrix represent the comparisons of each variable with itself, which does not make sense, that is why they are crossed out, indicating them in grey).

The predominance of green edges in the graph reflects mainly positive correlations. However, thinner red edges indicate negative relationships, such as between “Spr” and variables like “Alc” and “Ngo,” suggesting an inverse association between physical activity and substance use or nightlife behaviors. Overall, this network highlights the interplay among body image, sleep, and lifestyle variables, emphasizing strongly interrelated behavioral clusters. For further details on variable correlation, refer to the correlation matrix in Figure A1 (Appendix A).

### 3.6. Network Analysis of Nutritional and Sleep Variables

**Figure 4:**
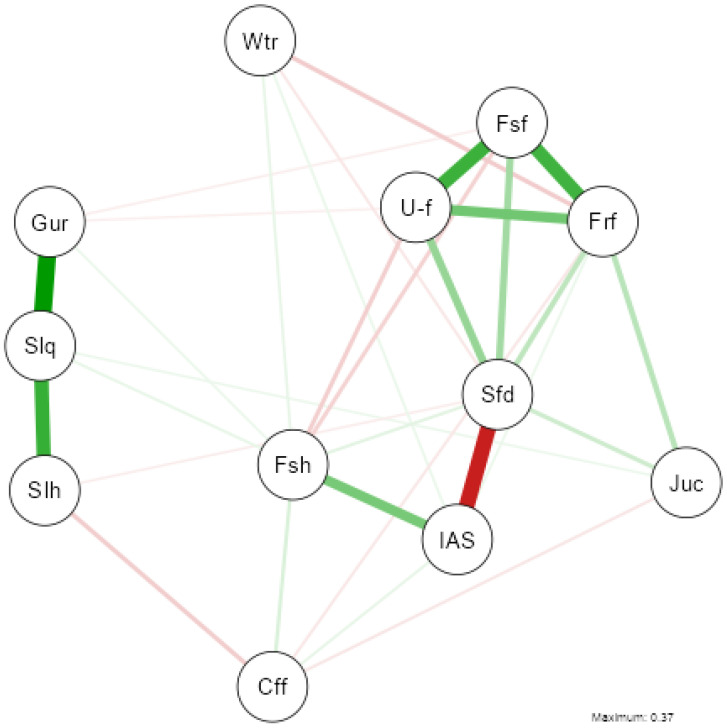
Gaussian Graphical Model of the network analysis between nutritional variables and sleep variables. (NOTE: The thickness of the lines reflects the magnitude of the relationship (partial correlation), while the colour indicates the direction: green for positive relationships and red for negative relationships).

The network analysis (Figure 4) of dietary habits and sleep variables reveals that sleep-related variables—”Gur” (Getting up rested), “Slq” (Sleep quality), and “Slh” (Sleeping hours)—form a strongly interconnected cluster, as indicated by thick green edges, reflecting strong positive associations. Similarly, “Fsf” (Fast food), “Frf” (Fried food), and “U-f” (Ultra-processed food) create a tightly connected group suggesting a pattern of dietary habits with positive relationships. “Fsh” (Fish consumption) exhibits a moderate positive connection with “Sfd” (Soft drinks consumption), implying that healthier food choices may be linked to greater dietary regulation.

**Figure A2:**
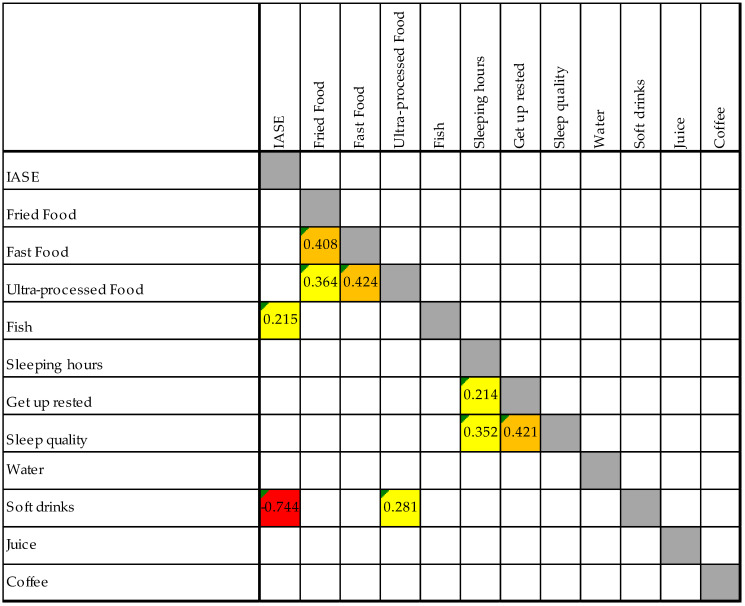
Spearman’s correlation for each pair of nutritional and sleep variables (White cells have no correlation (0.00–0.19); Yellow = Low correlation (0.20–0.39); Orange = Moderate correlation (0.40–0.59); Red = Strong correlation (≥0.60); Grey cells corresponding to the diagonal of the matrix represent the comparisons of each variable with itself, which does not make sense, that is why they are crossed out, indicating them in grey).

A red edge between “IAS” (IASE) and “Sfd” indicates a negative association, suggesting that IASE does not always align with soft drinks consumption. “Wtr” (Water intake) shows relatively weaker associations with other variables, indicating its lower integration into the primary dietary or sleep-related clusters. “Cff” (Coffee consumption) has moderate connections to both “Fsh” and “Slh,” suggesting its potential role in bridging dietary and sleep-related factors. For further details on variable correlations, refer to the correlation matrix in Figure A2 (Appendix A).

Finally, to assess whether the gender imbalance in the sample (with a clear majority of women over men) could affect the results, a Gaussian network analysis was performed separately for men and women. A comparison of the results is provided in Appendix B.

## 4. Discussion

This study examines the relationship between diet, lifestyle habits, and perceived sleep quality through a cross-sectional analysis. It explores how dietary factors, physical activity, and various socio-demographic aspects influence sleep quality.

While most participants report sleep durations within the recommended range (7–8 h), a significant proportion experiences moderate sleep quality and does not feel fully rested upon waking. This suggests that sleep quality is influenced by factors beyond duration. Previous studies have demonstrated that psychological stress [40], the use of electronic devices before bedtime [41], and a sedentary lifestyle [42] are associated with sleep disturbances. Additionally, psychological conditions such as anxiety and depression further compromise sleep and hinder both physical and mental recovery [43]. Addressing these factors is essential for improving sleep quality.

The analysis of socio-demographic variables reveals significant differences in sleep patterns. In terms of gender, women—despite sleeping slightly more than men—report lower sleep quality and less frequent feelings of being well-rested. This may be due to hormonal fluctuations and family responsibilities [44]. Regarding age, younger adults (18–30 years) tend to experience better sleep quality than older adults, aligning with literature indicating a decline in sleep satisfaction with age [45]. Education and income levels also correlate with sleep quality, and higher socioeconomic status is linked to better sleep outcomes. This trend is likely due to disparities in access to health-promoting resources [46,47,48,49,50].

Adequate sleep is strongly linked to better health outcomes, including lower BMI and healthier lifestyle habits [20]. Differences among participants with low (LSQ), medium (MSQ), and high (HSQ) sleep quality reveal a clear trend: as sleep quality improves, self-perceived health increases and BMI decreases, suggesting a beneficial effect on weight management.

Higher sleep quality correlates with healthier eating patterns, as indicated by a progressive increase in the Spanish Healthy Eating Index (IASE). Participants with better sleep consume more fish and water while reducing fried foods, fast food, ultra-processed products, and sugary drinks. These findings align with research emphasizing the role of balanced diets in improving sleep [51,52]. Sleep deprivation has been linked to increased appetite and higher consumption of calorie-dense foods, as demonstrated in several studies [26,53].

Another relevant aspect is the perception of waking up rested (YES_GUR). Participants who feel rested upon waking tend to follow a healthier diet, as evidenced by their greater adherence to the Mediterranean diet and higher fish consumption. They also exhibit a lower BMI compared to those who do not feel rested (NO_GUR). In particular, adherence to a balanced diet rich in essential nutrients, such as omega-3 fatty acids from fish, has been linked to improved sleep quality and a reduced risk of developing disordered eating behaviors [52].

Although reductions in sedentary behavior with better sleep quality were not statistically significant, physical activity levels consistently increased among participants with higher sleep quality, reinforcing previous findings [54].

Adequate sleep duration has been associated with higher energy levels, which could explain the increased physical activity observed among participants with sufficient sleep (SSH).

Good sleep quality enhances motivation for exercise and supports muscle recovery. The influence of sleep on physical activity is essential for improving overall physical well-being and maintaining a healthy body weight [55].

The relationship between sleep and physical activity is bidirectional: poor sleep reduces exercise motivation, while inactivity further disrupts sleep [25]. Additionally, the better-perceived health status and a higher level of physical activity among YES_GUR participants suggest that quality sleep is a predictor of overall well-being. Previous studies have highlighted that good sleep quality correlates with higher energy levels and a greater propensity for exercise, promoting an active and healthy lifestyle [56]. Interestingly, there is also a slight trend toward increased alcohol consumption in groups with better sleep, suggesting a possible connection between better sleep quality and greater socialization.

Regarding psychological factors, the study found that obesophobia, difficulty in self-control, and dissatisfaction with body image tend to decrease with improved sleep quality. This aligns with previous findings that highlight the positive impact of sleep on mental well-being and self-esteem [57]. Additionally, psychological factors such as sleep-related anxiety and dysfunctional beliefs about sleep can negatively affect perceived sleep quality independent of objective sleep measures [58,59].

Overall, improving sleep quality appears to have a significant positive effect on mental health, reducing the risk of anxiety and depression [60].

Two network analyses were conducted to better understand these relationships: one focusing on lifestyle and health factors and another on dietary habits. This approach allows for a detailed exploration of the variables influencing sleep quality.

The network analysis of lifestyle variables and sleep revealed a cluster comprising body image, obesophobia, Body Mass Index (BMI), and lack of self-control (Ncn), indicating a significant connection between weight concerns and sleep quality. Higher BMI and difficulties in weight control correlate with negative body image and poorer sleep quality. This relationship has been widely documented, with studies showing that obesity contributes to sleep disorders such as insomnia and sleep apnea [61,62]. Moreover, obesophobia can fuel stress cycles that interfere with sleep [63,64].

Another key finding is the strong positive association among sleep-related variables, such as sleep hours (Slh), sleep quality (Slq), and the perception of waking up rested (Gur). This aligns with research linking poor sleep to reduced psychophysical well-being [65].

The network analysis also highlights an association between alcohol (Alc), smoking (Smk), and night outings (Ngo), showing an inverse relationship with physical activity (Spr). Participants with higher alcohol consumption, smoking, and frequent night outings tend to engage in less physical activity. These behaviors have been linked to sedentary lifestyles and a negative impact on sleep quality [66,67,68,69].

The dietary network analysis further supports the association between healthy eating patterns and better sleep. Higher consumption of fish (Fsh) and water (Wtr) correlates with improved sleep quality (Slq, Slh, Gur), whereas a diet high in sugary drinks (Sfd) and ultra-processed foods is associated with poorer sleep [51]. Recent studies highlighted that a nutrient-rich diet, particularly one high in omega-3 fatty acids, vitamins, and minerals, can enhance both sleep duration and quality [70,71].

Interestingly, coffee consumption (Cff) shows a moderate association with sleep variables, suggesting individual differences in caffeine sensitivity. While caffeine is known to disrupt sleep [72], its effect depends on individual tolerance and timing of consumption [73].

### 4.1. Strengths and Limitations of the Study

The study’s strengths include its large sample size and the nationwide geographical representativeness of the Spanish population, allowing for a comprehensive analysis of socio-demographic, nutritional, and lifestyle factors influencing sleep quality. The use of the validated Nutritional and Social Healthy Habits Scale (NutSo-HH) ensures robust data collection. Additionally, integrating traditional statistical methods with network analysis enhances the study’s analytical rigor.

However, certain limitations must be acknowledged. The cross-sectional design of the study prevents the establishment of causal relationships between lifestyle factors and sleep quality. Additionally, self-reported data may introduce response and recall bias, potentially limiting generalizability. The use of snowball sampling may also lead to selection bias. The high proportion of female participants (~80%) may reflect a structural bias in health-related research participation [74,75,76]. Further biases concerning age and education level were identified, as the sample was predominantly young and highly educated.

This trend may be attributed to the dissemination of the questionnaire through social media, where younger individuals are more likely to engage with online surveys. To mitigate these biases, the research team is conducting a new wave of data collection to obtain a more balanced sample in terms of age, education level, and gender distribution. Finally, the study did not account for variables such as stress levels, sleep medication use, or other potential covariates that could influence the observed relationships. Future research should incorporate these factors, along with mental health indicators, to refine our understanding of these associations.

### 4.2. Public Health Implications of the Study

This study confirms that sleep quality is influenced by multiple factors, including lifestyle, dietary habits, socioeconomic status, and psychological well-being. The findings highlight the need for targeted public health interventions to improve sleep quality, particularly among vulnerable populations. While sleep duration is important, perceived sleep quality emerges as a key indicator of overall psychophysical well-being. Socio-demographic factors such as gender, age, and income significantly impact sleep quality, highlighting the need for targeted interventions. In particular, lower socioeconomic status and higher psychological distress are associated with poorer sleep quality, reinforcing the necessity of integrating these aspects into public health strategies. Future research should further explore these interactions to develop more effective targeted interventions.

To address these disparities, public health efforts should prioritize behavioral modifications that promote healthy sleep habits, especially in high-risk populations [77]. Tailored sleep hygiene education programs could be particularly effective, considering the socioeconomic and psychological barriers identified in this study. Moreover, national health initiatives, such as Spain’s Health Promotion and Prevention Strategy (SHPP) [78], developed by the Ministry of Health, could incorporate sleep quality as a key component, addressing both physical and mental health aspects. Similar programs in other countries, such as Healthy People 2030 in the United States, have demonstrated the value of multi-faceted strategies for improving sleep [79]. Public health policies should, therefore, prioritize sleep as a crucial determinant of health and implement targeted interventions to reduce the impact of socioeconomic disparities. These could include community-based programs offering accessible sleep education, workplace policies supporting better sleep practices, and mental health initiatives aimed at stress reduction and addressing anxiety and depression. By integrating these measures, policymakers can enhance sleep quality, ultimately improving overall health outcomes and quality of life.

## 5. Conclusions

This study underscores the role of socio-demographic factors, lifestyle choices, and physical activity in shaping sleep quality. The findings reinforce the importance of a balanced diet, regular exercise, and stress management in promoting restorative sleep. Future research should focus on longitudinal studies to establish causal relationships and inform evidence-based policies for improving sleep health at the population level.

## Figures and Tables

**Table 1:** Sample socio-demographic characteristics (N = 22,181).

		Mean (SD) or N (%)
Sex	Male	4251 (19.2%)
	Female	17,930 (80.8%)
Age (years)	Age total	34.9 (11.7)
	Age male	36.5 (13.4)
	Age female	34.5 (11.2)
	Young (≤30 years)	9692 (43.7%)
	Adults (>30 years)	12,489 (56.3%)
Education level	Basic Education	7027 (31.7%)
	Higher Education	15,154 (68.3%)
Income level	Low	9727 (43.9%)
	Medium–high	10,616 (47.9%)
	No answer	1839 (8.3%)
Municipality size	<2000	1014 (4.6%)
	2000–10,000	3587 (16.2%)
	>10,000	17,580 (79.3%)
Living arrangement	Living alone	2202 (9.9%)
	Not living alone	19,979 (90.1%)
Family life	Living with family	16,732 (75.4%)
	Living without family	5449 (24.6%)

**Table 2:** Socio-demographic differences in sleep variables.

Socio-Demographic Variables		Sleep Hours		Get Up Rested		Sleep Quality	
		Mean (SD)	*p*-Value ^$^	Mean (SD)	*p*-Value ^$^	Mean (SD)	*p*-Value ^$^
Sex	Male	2.48 (0.73)	<0.001	2.61 (0.58)	<0.001	3.48 (1.03)	<0.001
	Female	2.53 (0.73)		2.53 (0.59)		3.36 (0.96)	
Age	Young (18–30 years)	2.66 (0.70)	<0.001	2.54 (0.57)	0.406	3.51 (0.98)	<0.001
	Adults (>30 years)	2.41 (0.74)		2.54 (0.59)		3.29 (1.04)	
Education	Basic Education	2.49 (0.76)	<0.001	2.50 (0.60)	<0.001	3.30 (1.05)	<0.001
	Higher Education	2.53 (0.72)		2.56 (0.58)		3.42 (1.00)	
Incomes	Low	2.51 (0.75)	0.562	2.51 (0.59)	<0.001	3.32 (1.03)	<0.001
	Medium–high	2.51 (0.71)		2.57 (0.57)		3.43 (1.00)	
Municipality size	<2000	2.53 (0.73)	0.076 ^&^	2.52 (0.58)	0.061 ^&^	3.35 (1.02)	0.065 ^&^
	2000–10,000	2.56 (0.77)		2.52 (0.62)		3.36 (1.06)	
	>10,000	2.51 (0.73)		2.55 (0.58)		3.39 (1.01)	
Living arrangement	Living alone	2.52 (0.73)	<0.001	2.54 (0.58)	0.458	3.39 (1.01)	0.253
	Not living alone	2.45 (0.75)		2.55 (0.59)		3.36 (1.02)	
Family life	Living with family	2.54 (0.72)	0.001	2.55 (0.58)	0.207	3.44 (0.99)	<0.001
	Living without family	2.51 (0.74)		2.54 (0.58)		3.37 (1.02)	

^$^ Mann–Whitney U Test; ^&^ Kruskal–Wallis Test.

**Table 3:** (**a**) Relationship between nutrition/lifestyle and sleep hours. (**b**) Relationship between nutrition/lifestyle and getting up rested.

(**a**)			
**Numerical Variable**	**Sleep Hours**		
	**Insufficient Sleep Hours (ISH)**	**Sufficient Sleep Hours (SSH)**	
	**Median (IQR)**	**Median (IQR)**	***p*-Value ^$^**
IASE	55.0 (13.5)	56.0 (12.6)	<0.001
BMI (kg/m^2^)	23.5 (5.66)	22.6 (4.72)	<0.001
Fried food	2.00 (1.00)	2.00 (1.00)	0.286
Fast food	2.00 (1.00)	2.00 (1.00)	0.399
Ultra-processed food	2.00 (1.00)	2.00 (1.00)	0.172
Fish	2.00 (0.50)	2.00 (0.50)	0.901
Water	3.00 (1.00)	3.00 (1.00)	<0.001
Sugary soft drinks	1.00 (1.00)	1.00 (1.00)	<0.001
Juice	1.00 (1.00)	1.00 (1.00)	0.887
Coffee and energy drinks	2.00 (1.00)	2.00 (1.00)	<0.001
Sedentary lifestyle	1.00 (1.00)	1.00 (1.00)	<0.001
Self-perceived health	4.00 (1.00)	4.00 (1.00)	<0.001
Sport (minuts/week)	90.0 (225)	135.0 (225)	<0.001
Smoking	1.00 (1.00)	1.00 (1.00)	<0.001
Alcohol	2.00 (1.00)	2.00 (1.00)	0.017
Getting drunk	1.00 (1.00)	1.00 (1.00)	0.556
Night outings	1.00 (1.00)	1.00 (1.00)	<0.001
Obesophobia	4.00 (2.00)	3.00 (1.00)	<0.001
No control	3.00 (2.00)	3.00 (1.00)	<0.001
Body image	4.00 (2.00)	3.00 (1.00)	<0.001
(**b**)			
**Numerical Variable**	**Get Up Rested**		
	**NO Get Up Rested (NO_GUR)**	**YES Get Up Rested (YES_GUR)**	
	**Median (IQR)**	**Median (IQR)**	***p*-Value ^$^**
IASE	55.0 (12.5)	56.0 (12.5)	<0.001
BMI (kg/m^2^)	23.2 (5.57)	22.9 (4.80)	<0.001
Fried food	2.00 (1.00)	2.00 (1.00)	<0.001
Fast food	2.00 (1.00)	2.00 (1.00)	<0.001
Ultra-processed food	2.00 (1.00)	2.00 (1.00)	<0.001
Fish	2.00 (0.50)	2.00 (0.50)	<0.001
Water	3.00 (1.00)	3.00 (1.00)	0.008
Sugary soft drinks	1.00 (1.00)	1.00 (1.00)	<0.001
Juice	1.00 (1.00)	1.00 (1.00)	0.954
Coffee and energy drinks	2.00 (1.00)	2.00 (1.00)	0.704
Sedentary lifestyle	1.00 (1.00)	1.00 (1.00)	<0.001
Self-perceived health	4.00 (1.00)	4.00 (0.00)	<0.001
Sport (minutes/week)	90.0 (210)	135.0 (225)	<0.001
Smoking	1.00 (0.00)	1.00 (0.00)	<0.001
Alcohol	1.00 (1.00)	2.00 (1.00)	<0.001
Getting drunk	1.00 (0.00)	1.00 (0.00)	0.732
Night outings	1.00 (0.00)	1.00 (0.00)	0.005
Obesophobia	4.00 (3.00)	4.00 (2.00)	<0.001
No control	3.00 (2.00)	2.00 (1.00)	<0.001
Body image	4.00 (2.00)	3.00 (1.00)	<0.001

Note: ^$^ = Mann–Whitney U Test; IASE = Spanish Healthy Eating Index; BMI = Body Mass Index; IQR = Inter-quartile Range.

**Table 4:** Relationship between nutrition and lifestyle variables and perceived quality of sleep.

Numerical Variable	Low Sleep Quality (LSQ)	Medium Sleep Quality (MSQ)	High Sleep Quality (HSQ)	*p*-Value ^&^
	Median (IQR)	Median (IQR)	Median (IQR)	
IASE	52.0 (15.0)	55.0 (13.5)	56.0 (12.5)	LSQ-MSQ (*p* < 0.001)
				LSQ-HSQ (*p* < 0.001)
				MSQ-HSQ (*p* < 0.001)
BMI	24.0 (6.96)	23.2 (5.39)	22.8 (4.73)	LSQ-MSQ (*p* < 0.001)
				LSQ-HSQ (*p* < 0.001)
				MSQ-HSQ (*p* < 0.001)
Fried food	2.00 (1.00)	2.00 (1.00)	2.00 (1.00)	LSQ-MSQ (*p* = 0.101)
				LSQ-HSQ (*p* = 0.013)
				MSQ-HSQ (*p* = 0.124)
Fast food	2.00 (1.00)	2.00 (1.00)	2.00 (1.00)	LSQ-MSQ (*p* < 0.001)
				LSQ-HSQ (*p* < 0.001)
				MSQ-HSQ (*p* < 0.001)
Ultra-processed food	2.00 (1.00)	2.00 (1.00)	2.00 (1.00)	LSQ-MSQ (*p* = 0.033)
				LSQ-HSQ (*p* < 0.001)
				MSQ-HSQ (*p* < 0.001)
Fish	2.00 (0.50)	2.00 (0.50)	2.00 (0.50)	LSQ-MSQ (*p* = 0.003)
				LSQ-HSQ (*p* < 0.001)
				MSQ-HSQ (*p* = 0.011)
Water	3.00 (1.00)	3.00 (1.00)	3.00 (1.00)	LSQ-MSQ (*p* = 0.148)
				LSQ-HSQ (*p* = 0.002)
				MSQ-HSQ (*p* < 0.001)
Sugary soft drinks	1.00 (1.00)	1.00 (1.00)	1.00 (1.00)	LSQ-MSQ (*p* = 0.005)
				LSQ-HSQ (*p* < 0.001)
				MSQ-HSQ (*p* < 0.001)
Juice	1.00 (1.00)	1.00 (1.00)	1.00 (1.00)	LSQ-MSQ (*p* = 0.984)
				LSQ-HSQ (*p* = 0.235)
				MSQ-HSQ (*p* < 0.001)
Coffee and energy drinks	2.00 (1.00)	2.00 (1.00)	2.00 (1.00)	*p* = 0.212
Sedentary lifestyle	1.00 (1.00)	1.00 (1.00)	1.00 (1.00)	*p* = 0.089
Self-perceived health	3.00 (2.00)	4.00 (1.00)	4.00 (1.00)	LSQ-MSQ (*p* < 0.001)
				LSQ-HSQ (*p* < 0.001)
				MSQ-HSQ (*p* < 0.001)
Sport	30.0 (203)	105.0 (225)	135.0 (225)	LSQ-MSQ (*p* < 0.001)
				LSQ-HSQ (*p* < 0.001)
				MSQ-HSQ (*p* < 0.001)
Smoking	1.00 (0.00)	1.00 (0.00)	1.00 (0.00)	LSQ-MSQ (*p* < 0.001)
				LSQ-HSQ (*p* < 0.001)
				MSQ-HSQ (*p* < 0.001)
Alcohol	1.00 (1.00)	2.00 (1.00)	2.00 (1.00)	LSQ-MSQ (*p* < 0.001)
				LSQ-HSQ (*p* < 0.001)
				MSQ-HSQ (*p* < 0.001)
Getting drunk	1.00 (0.00)	1.00 (0.00)	1.00 (0.00)	*p* = 0.505
Night outings	1.00 (0.00)	1.00 (0.00)	1.00 (0.00)	LSQ-MSQ (*p* < 0.001)
				LSQ-HSQ (*p* < 0.001)
				MSQ-HSQ (*p* < 0.001)
Obesophobia	4.00 (2.00)	4.00 (2.00)	3.00 (2.00)	LSQ-MSQ (*p* < 0.001)
				LSQ-HSQ (*p* < 0.001)
				MSQ-HSQ (*p* < 0.001)
No control	3.00 (3.00)	3.00 (2.00)	2.00 (1.00)	LSQ-MSQ (*p* < 0.001)
				LSQ-HSQ (*p* < 0.001)
				MSQ-HSQ (*p* < 0.001)
Body image	4.00 (2.00)	4.00 (2.00)	3.00 (1.00)	LSQ-MSQ (*p* < 0.001)
				LSQ-HSQ (*p* < 0.001)
				MSQ-HSQ (*p* < 0.001)

Note: ^&^ = Kruskal–Wallis Test with Dwass–Steel–Critchlow–Fligner (DSCF) method; IASE = Spanish Healthy Eating Index; BMI = Body Mass Index; IQR = Inter-quartile Range.

**Figure A3:**
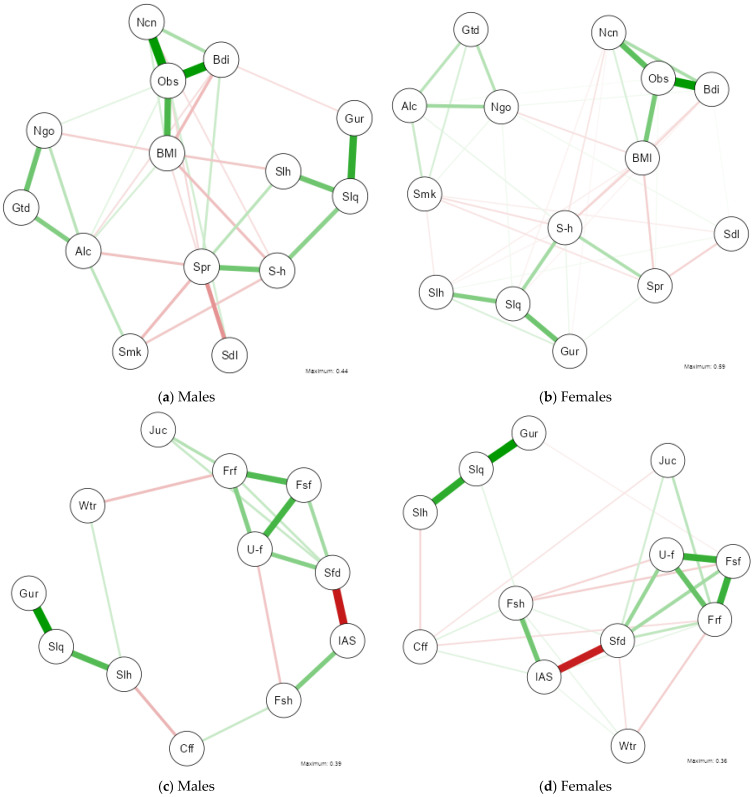
(**a**–**d**). Comparison between men and women of the Gaussian Graphical Model of network analysis. [(**a**) Gaussian graphical model of network analysis between health and lifestyle variables and sleep variables for men; (**b**) Gaussian graphical model of network analysis between health and lifestyle variables and sleep variables for women; (**c**) Gaussian graphical model of network analysis between nutritional variables and sleep variables for men; (**d**) Gaussian graphical model of network analysis between nutritional variables and sleep variables for women]. (NOTE: The thickness of the lines reflects the magnitude of the relationship (partial correlation), while the colour indicates the direction: green for positive relationships and red for negative relationships).

## Data Availability

The data presented in this study are available from the corresponding author upon reasonable request.

## Abbreviations

The following abbreviations are used in this manuscript:

ISH	Insufficient Sleep Hours
SSH	Sufficient Sleep Hours
NO_GUR	NO Get Up Rested
YES_GUR	YES Get Up Rested
LSQ	Low Sleep Quality
MSQ	Medium Sleep Quality
HSQ	High Sleep Quality
Bdi	Body image
Obs	Obesophobia
Ncn	No control
Slh	Sleeping hours
Slq	Sleep quality
Gur	Get up rested
Alc	Alcohol
Smk	Smoking
Ngo	Night outings
Spr	Sport
Fsf	Fast food
Frf	Fried food
U-f	Ultra-processed food
Fsh	Fish consumption
Sfd	Soft drinks consumption
IAS	IASE
Wtr	Water intake
Cff	Coffee consumption

## Appendix B

A comparison between men and women was conducted using the Gaussian Graphical Model of network analysis (Figure A3a–d) to examine the relationships between nutrition and sleep variables, as well as between lifestyle and sleep variables.

Although the graphical representations are arranged differently, the connections between the nodes remain largely unchanged. Variables that exhibited a strong dependence in the total population sample (Figure 3 and Figure 4) maintained this relationship in both the male-only (Figure A3a,c) and female-only (Figure A3b,d) samples. This finding suggests that, despite the imbalance in sex distribution within the sample, the results obtained provide a reliable approximation of the trends that may occur in the Spanish population.
